# Neotropical stingless bees display a strong response in cold tolerance with changes in elevation

**DOI:** 10.1093/conphys/coac073

**Published:** 2022-12-21

**Authors:** Victor H Gonzalez, Kennan Oyen, Nydia Vitale, Rodulfo Ospina

**Affiliations:** Undergraduate Biology Program and Department of Ecology and Evolutionary Biology, University of Kansas, Lawrence, KS, 66045, USA; Department of Biological Sciences, McMicken College of Arts and Sciences, University of Cincinnati, 318 College Drive, Cincinnati, OH, 45221, USA; Instituto Argentino de Investigaciones de las Zonas Áridas, CONICET, Mendoza, 5500, Argentina; Laboratorio de Investigaciones en Abejas, Universidad Nacional de Colombia, Santa Fé de Bogotá, 111321, Colombia

**Keywords:** Colombia, meliponiculture, physiological thresholds, sustainability

## Abstract

Tropical pollinators are expected to experience substantial effects due to climate change, but aspects of their thermal biology remain largely unknown. We investigated the thermal tolerance of stingless honey-making bees, the most ecologically, economically and culturally important group of tropical pollinators. We assessed changes in the lower (CT_Min_) and upper (CT_Max_) critical thermal limits of 17 species (12 genera) at two elevations (200 and 1500 m) in the Colombian Andes. In addition, we examined the influence of body size (intertegular distance, ITD), hairiness (thoracic hair length) and coloration (lightness value) on bees’ thermal tolerance. Because stingless beekeepers often relocate their colonies across the altitudinal gradient, as an initial attempt to explore potential social responses to climatic variability, we also tracked for several weeks brood temperature and humidity in nests of three species at both elevations. We found that CT_Min_ decreased with elevation while CT_Max_ was similar between elevations. CT_Min_ and CT_Max_ increased (low cold tolerance and high heat tolerance) with increasing ITD, hair length and lightness value, but these relationships were weak and explained at most 10% of the variance. Neither CT_Min_ nor CT_Max_ displayed significant phylogenetic signal. Brood nest temperature tracked ambient diel variations more closely in the low-elevation site, but it was constant and higher at the high-elevation site. In contrast, brood nest humidity was uniform throughout the day regardless of elevation. The stronger response in CT_Min_, and a similar CT_Max_ between elevations, follows a pattern of variation documented across a wide range of taxa that is commonly known as the Brett’s heat-invariant hypothesis. Our results indicate differential thermal sensitivities and potential thermal adaptations to local climate, which support ongoing conservation policies to restrict the long-distance relocations of colonies. They also shed light on how malleable nest thermoregulation can be across elevations.

## Introduction

Pollinators supply essential ecosystem services and bees (~20 000 spp.) are widely recognized as the most important pollinators of wild and cultivated plants (Klein *et al.*, 2007; Michener, 2007). However, bees have already experienced changes in community composition, population vigor, distribution and interactions with host plants due to landscape-level alterations and climate change (e.g. Bartomeus *et al.*, 2013; Kerr *et al.*, 2015). Thus, forecasting bees’ responses to these environmental stressors is imperative to anticipate potential impacts on ecosystem function (Scheffers *et al.*, 2016; Halsch *et al.*, 2021), and, ultimately, to develop strategies that mitigate the effects on agriculture and food security.

Insects, as ectotherm organisms, are most vulnerable to climate change, particularly those from tropical areas where the effects are expected to be substantial due to organisms living close to their maximum tolerable temperature and limited acclimation capacities (Deutsch *et al.*, 2008; Kingsolver *et al.*, 2013). However, information on the thermal biology of tropical insects, including those of ecological and economic importance such as bees, is still limited. Thus, in this study, we were interested in assessing the thermal tolerance of neotropical stingless bees (Apidae: Meliponini) using their Critical Thermal Limits, the minimum (CT_Min_) and maximum (CT_Max_) temperatures at which an animal can maintain muscle control (Lutterschmidt and Hutchison, 1997). These physiological traits are measured under controlled conditions in the laboratory and are key for our understanding of an organism’s ecology and evolution, as well as the responses to changes in climate and land use (Angilletta, 2009; Sunday *et al.*, 2011). For example, changes in critical thermal limits have been associated with variations in some aspects of climate, such as precipitation and temperature, which determine species’ distribution at both geographic and temporal gradients (e.g. Sunday *et al.*, 2011; Kellermann *et al.*, 2012; García-Robledo *et al.*, 2018; Nascimento *et al.*, 2022). Given that critical thermal limits are good predictors of an organism’s potential response to extreme temperature changes, they are commonly used in calculating thermal sensitivity indices, which estimates a population or species’ susceptibility to climate change (Deutsch *et al.*, 2008; Sunday *et al.*, 2014; Clusella-Trullas *et al.*, 2021; Roeder *et al.*, 2021a).

Considering that thermal limits estimates may vary in response to abiotic and biotic factors (e.g. Roeder *et al.*, 2021b; Nascimento *et al.*, 2022), the geographic distribution and the morphological and biological diversity of stingless bees (see below) makes them an excellent model system to explore the influence of these potential covariates on their thermal tolerance traits. For example, in some insects including bees, CT_Max_ decreases with increasing elevation (García-Robledo *et al.*, 2016; Oyen *et al.*, 2016; Gonzalez *et al.*, 2020) and with age and starvation (Nyamukondiwa and Terblanche, 2009; Chidawanyika *et al.*, 2017). However, CT_Max_ may increase with increasing body size (Baudier *et al.*, 2018) and with acute exposure to pesticides (Gonzalez *et al.*, 2022b). These responses may vary depending on the species, community or taxonomic group, as elevation, age, starvation or body size does not influence estimates of CT_Max_ in some ants (Bishop *et al.*, 2017) and bees (Hamblin *et al.*, 2017; Oyen and Dillon, 2018; Gonzalez *et al.*, 2020, 2022a, 2022c). Thus, we were also interested in determining the effect of morphological traits such as body size, hairiness and color, as well as of elevation on stingless bees’ thermal tolerance.

Some species of stingless bees have already been categorized as threatened or vulnerable to extinction (Nates-Parra, 2007; dos Santos *et al.*, 2021) and predictions based on niche modeling studies under climate change scenarios suggest significant reductions (up to 70%) in bees’ climatically suitable areas across South America (Giannini *et al.*, 2012, 2017, 2020; Gonzalez *et al.*, 2021). In addition, while the interest in stingless bees keeping (meliponiculture) as an environmentally sustainable and poverty alleviating practice has increased, the potential extinction risk for natural populations has also grown. The intensive extraction and long-distance relocation of wild nests to areas outside of bees’ native range with unsuitable habitats, including changes in elevation, might not only increase the spread of parasites and pathogens but has already resulted in low rates of colony establishment or total loss (Gonzalez *et al.*, 2021; dos Santos *et al.*, 2022). In addition, nest relocation might alter the genetic structure of both wild and managed populations (Byatt *et al.*, 2016; Chapman *et al.*, 2018). Thus, considering that thermal limits determine species’ fundamental niche and have a strong influence on the species potential distribution (Angilletta, 2009; Sunday *et al.*, 2011), information on stingless bees’ thermal tolerance might improve our predictions of their responses to anthropogenic change and inform conservation practices and policies.

Herein, we assessed the lower and upper thermal limits for 17 species (12 genera) of stingless bees at two elevations (200 and 1500 m) in the Colombian Andes. Because temperature decreases with elevation, we predict that bees at high elevation will display lower CT_Min_ and CT_Max_ (greater cold tolerance and lower heat tolerance) than bees from low elevations. Small bees cool down and heat up more quickly than large bees because of their high surface area to volume ratio, which increases convective heat transfer (Heinrich and Heinrich, 1983; Oyen *et al.*, 2016). Thus, we expect that CT_Min_ decreases while CT_Max_ increases (higher cold and heat tolerance) with increasing body size. Because body hair may form an insulation layer that mitigates heat loss and increases retention of cool air (Peters *et al.*, 2016; Buxton *et al.*, 2021), we expect CT_Min_ to decrease and CT_Max_ to increase with increasing hairiness. Because dark integument improves heat gain and increases resistance to ultraviolet radiation (Bishop *et al.*, 2016; Law *et al.*, 2020), we expect CT_Min_ to decrease and CT_Max_ to increase with increasing darker color.

Finally, plastic responses in thermal tolerance may result from the thermal environment in which the immature stages developed, and such responses are critical in the context of global warming because they can potentially compensate for the negative consequences of expected changes in environmental conditions (Kellermann and van Heerwaarden, 2019). Given the diversity of stingless bees’ nesting biology, it is reasonable to assume that the thermal environment of their immature stages also varies significantly. Unfortunately, thermal studies of stingless bees’ nests are limited. Available studies suggest that stingless bees are poor thermoregulators of their nests in comparison to honey bees (Roubik, 2006; Michener, 2007) and that, at least in some species, hygroregulation (regulation of humidity) is more important than thermoregulation for colony health (Ayton *et al.*, 2016). Understanding this aspect of the stingless bees’ thermal biology is also important for their management and conservation, as beekeepers often relocate colonies across the altitudinal gradient. Thus, as an initial attempt to fill this gap in knowledge and to explore potential social responses to climatic variability, we also tracked changes in the brood temperature and humidity in nests of three species in relation to ambient conditions at both elevations. If stingless bees are both poor thermoregulators and hygroregulators, we predict that nests at high elevations in cool, humid habitats would display mean lower internal temperature and higher relative humidity when compared to nests in hot, dry lowland habitats. Alternatively, if stingless bees are good hygroregulators but poor thermoregulators, we predict differences in nest temperature only.

## Material and Methods

### Study organisms

**Figure 1:**
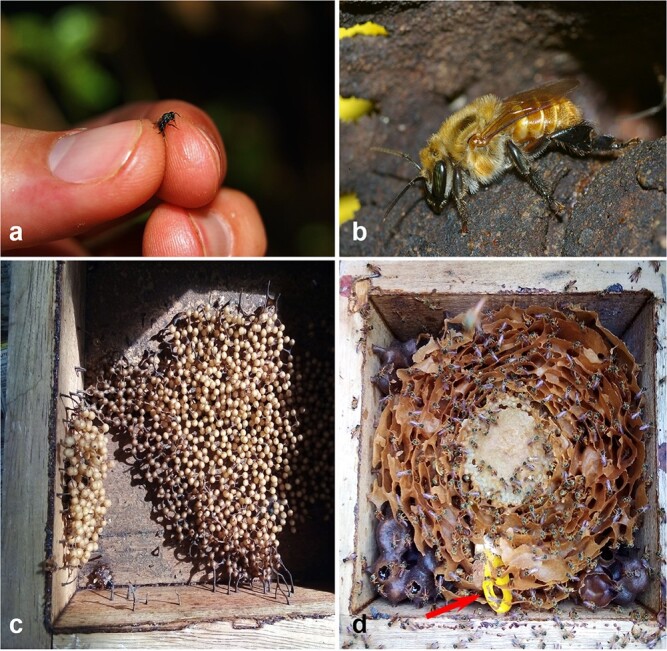
Body size and internal nest architecture of stingless bees. (**a**) Small worker of *Plebeia* sp. (**b**) Worker of *Melipona eburnea*, about the size of the European honey bee (credits: C. Rasmussen). (**c**) Brood cells of *F. paupera* arranged in clusters and not protected by an involucrum (layer of mixture of wax and resin). (**d**) Brood cells of *T. angustula* located in the center of nest and protected by layers of involucrum (upper layers removed for photography). Data logger placed in a yellow plastic holder is indicated by the red arrow.

Stingless bees are social, pollen generalist, honey-making bees restricted to the tropics. They live in perennial colonies and pollinate a broad array of native and cultivated plants, including global commodities such as coffee (e.g. Cauich *et al.*, 2006; Slaa *et al.*, 2006; Michener, 2007). They have traditionally been used by Indigenous and non-Indigenous people to obtain honey, pollen, cerumen and propolis for diverse purposes, including food, medicine and crafts (Gonzalez *et al.*, 2018; Quezada-Euán *et al.*, 2018). There are more than 500 species of stingless bees, most of them (80%) inhabiting the American tropics that range tremendously in body size, color, body shape, hairiness and nesting biology. Some species are minute (2–4 mm), black or yellow with narrow and bare bodies, while others are reddish with robust and hairy bodies, as large as or larger than the European honey bee (Fig 1a,b). Many species nest inside pre-existing cavities in the ground, tree trunks or fabricated constructions, while others build aerial nests or inside the nests of living termites. Internally, brood cells are either in clusters or in combs, often surrounded by several layers of a mixture of wax and resin (involucrum) (Michener, 2007) (Fig 1c,d). In addition, stingless bees occur in a wide range of habitats and ecosystems including urban areas, from sea level up to 4000 m in the Andes, and from tropical rain and dry forests to cloud forests (Gonzalez and Engel, 2004).

### Study locations and bee collections

We conducted field and experimental work during the dry season (January to April 2021) at two elevations on the western slope of the Oriental cordillera in Colombia (Department of Cundinamarca): Beltrán, a municipality within the tropical dry forest ecosystem along the Magdalena River (4°48.020’N, 74°44.394’W, 237 m, hereon low-elevation site) and San Antonio del Tequendama (4°38.107’N, 74°21.331’W, 1581 m, hereon high-elevation site), a municipality situated within montane cloud forest (Supplementary Fig S1). As it is common in the Andean region of Colombia, both locations are characterized by anthropogenically transformed habitats, such as open areas for agriculture and cattle ranching, with patches of secondary vegetation. We captured bees from managed hives kept by beekeepers, as well as from wild nests that we found with the assistance of local consultants. Some species of stingless bees, such as those in the genera *Oxytrigona* Cockerell and *Scaptotrigona* Moure, are highly defensive and may display suicidal biting (Shackleton *et al.*, 2015). Consequently, bees captured from nests that were unintentionally provoked resulted in high mortality prior the start of the experiments. Thus, we avoided disturbing nests and only collected returning forager bees at the nest entrance, usually between 9:00 and 11:00 h, with the aid of an insect net. We then transferred bees individually to a plastic vial, which we then capped with fabric (~1 mm mesh), and fed them *ad libitum* with a drop of 50% sucrose solution placed at the bottom of the vial. We kept bees inside a Styrofoam cooler with an ice pack covered in a piece of cloth (16–19°C) until we completed fieldwork. We tested bees within 1–2 h after captured in the field.

### Ambient temperature and humidity

At each location, we measured ambient temperature and humidity using iButton data loggers (weight: 3.104 g; DS1923 Hygrochron™; Maxim Integrated, San Jose, California), which we protected from solar radiation with aluminum foil and hung at 1 m above ground from tree branches (See Gonzalez *et al.*, 2020). We recorded temperature and humidity every 30 min for seven consecutive weeks.

### Critical thermal limits assays

We measured bees’ heat and cold tolerances using a dynamic (ramping temperature) protocol with the Elara 2.0 (IoTherm, Laramie, WY), a portable fully programmable heating/cooling anodized aluminum stage designed for precision temperature control of laboratory and field samples. The stage was modified with a Styrofoam cooler and clear acrylic lid to minimize the impact of airflow across the aluminum sample stage and maintain temperature stability across all vials. We placed bees individually inside glass vials (12 × 35 mm, 1.85 cm^3^) and plugged them with a moistened cotton ball (~ 0.2 mL of distilled water per cotton ball) to ensure enough humidity during the assays. We used an initial temperature of 22°C and held bees for 10 minutes at this temperature before increasing it or decreasing it at a rate of 0.5°C min^−1^. We placed vials horizontally on the stage to avoid bees from climbing along the vial. To estimate the temperature inside the vials, we placed a K-type thermocouple inside two empty glass vials plugged with a cotton ball. We individually tracked these vial temperatures using a TC-08 thermocouple data logger (Pico Technology, Tyler, TX, USA). As an approximation of bees’ thermal limits, we used the temperature at which bees show signs of curling (CT_Min_, Oyen and Dillon, 2018) or lost muscular control, spontaneously flipping over onto their dorsa and spasming (CT_Max_, Lutterschmidt and Hutchison, 1997; García-Robledo *et al.*, 2016, 2018). Then, after these bioassays concluded, we used specimens to measure morphological traits as indicated below.

### Body size, hairiness and integumental color

We estimated body size by measuring the minimum intertegular distance (ITD) (Cane, 1987) of each specimen. Given that the flight muscles involved in endogenous heat production are in the thorax, and hairs are generally short and sparse on the disc of mesoscutum, we measured the maximum hair length along the anterolateral corners of mesoscutum as a proxy of body hairiness. We measured hair length in 10 specimens per species that we randomly selected from each elevation and used average values in the analyses. We took these measurements using an ocular micrometer on an S6E stereomicroscope (Leica Microsystems, Wetzlar, Germany). As in other studies assessing coloration of the insect’s cuticle (e.g. De Souza *et al.*, 2020; Law *et al.*, 2020), we estimated body luminance by measuring lightness value (*L*) on the disc of mesoscutum, which is a component of the hue, saturation and lightness (HSL) digital color model. The lightness value represents the overall darkness or lightness of a color, and it ranges from 0 to 100%, with 0 being black and 100 white. We used digital images of the dorsum of five specimens per species randomly selected from each elevation and used the average value. Using a standardized gray background, we generated images with the Macropod Pro 3D (www.macroscopicsolutions.com). Then, we used the eyedropper tool with a 31 by 31 average pixel radius in Adobe Photoshop (Adobe, San Jose, CA) to sample the RGB color model (red, green, blue) and then transformed them to a HSL color model (Koch *et al.*, 2014).

Voucher specimens are in the Laboratorio de Abejas of the Universidad Nacional de Colombia, Santa Fé de Bogotá and in the Division of Entomology, University of Kansas Natural History Museum (Biodiversity Institute), Lawrence, Kansas.

### Brood temperature and humidity

We monitored brood temperature and humidity in wooden boxes containing managed hives of *Frieseomelitta paupera* (Provancher) at the low-elevation site (two hives; dimensions = 30.0 × 24.5 × 25.0 cm, ~ 1.7 cm thick), *Melipona eburnea* Friese at the high-elevation site (one hive; dimensions = 30.0 × 24.5 × 25.0 cm, ~ 1.7 cm thick) and *Tetragonisca angustula* (Illiger) at both elevations (one hive at each elevation; dimensions = 32.5 × 17.5 × 17.5 cm, ~ 1.7 cm thick). In Colombia, the first species is restricted to dry forests, the second species typically occurs at mid elevations and the third species is widely distributed across the elevation gradient, from sea level up to 1800 m (Nates-Parra and Londoño, 2013). To facilitate comparisons, we chose colonies about the same weight, as suggested for assessing condition of honey bee and stingless bee hives (Ayton *et al.*, 2016). We chose these species because they differ in their internal nest architecture, they are commonly used in meliponiculture in Colombia (Nates-Parra and Londoño, 2013) and they were available to us for study at each location by local beekeepers. *Frieseomelitta paupera* builds brood cells in clusters and without an involucrum (Fig 1c) while the other two species build cells in combs and surrounded by several layers of involucrum (as in Fig 1d). Thus, based on nest architecture alone, we expected differences in the bees’ ability to regulate brood temperature and humidity passively. We inserted an iButton data logger inside the brood chamber (Fig 1d), which we placed in a plastic holder (Thermochron Fob) wrapped in a net mesh to prevent bees from covering it with propolis, and continuously recorded temperature and humidity every 30 min. To estimate the effect of the thermal insulation of the box material on intranidal temperature and humidity, at each location and as a control, we placed a data logger prepared as above inside an empty wooden artificial hive (30.0 × 24.5 × 25.0 cm, ~ 1.7 cm thick), located next (50–100 cm) to the experimental hives. Simultaneously, at each location and during the study period, we recorded ambient temperature and humidity as indicated above. We set up these data loggers on January 29 at the high-elevation site and on February 6 at the low-elevation site, but we only analyzed the data recorded a week after their placement inside the hives to allow bees to recover from the manipulation. Due to equipment limitations, we were only able to measure brood temperature for *F. paupera* between February 6 and March 29 but measured both brood temperature and humidity between March 29 and April 20. According to beekeepers, all nests have been established for at least one year prior to our observations.

### Statistical analysis

We conducted statistical analyses in R (R Core Team, 2018). To test for differences in the daily air temperatures and relativity humidity between elevations, we used a one-way ANOVA model using the lm function. To test for differences in ITD, hair length and lightness value among species and between elevations, we implemented a linear mixed-effect model (LMM) using the lmer function in the lme4 package (Bates *et al.*, 2015) with species and elevation (low and high) as fixed factors and nest identity as a random factor. To assess for differences in ITD for each of the three species that were collected at both elevations (*Scaptotrigona magdalenae* Engel, *T. angustula* and *Trigona fulviventris* Guérin-Méneville), we implemented a similar LMM for each species individually. To evaluate the relationship between CT_Min_ and CT_Max_, as well as between each morphological trait and CT_Min_ and CT_Max_, we implemented a linear regression analysis using the lm function. To compare the slope of regression between elevations, we specified a model that included the interaction between CT_Max_ and elevation (low and high) and between each morphological trait and elevation. We used a mixed-model ANCOVA to compare CT_Min_ and CT_Max_ between elevations while controlling for the effects of each morphological trait. We implemented a LMM with elevation and species as fixed factors, a morphological trait as covariate and nest identity as a random nested variable within species. To assess the relative importance of morphological traits on critical thermal limits, we first implemented a linear model with either CT_Min_ or CT_Max_ as the response variable and all morphological traits as predictors. Then, we used the function stepAIC from the MASS package (Venables and Ripley, 2002) to select the model with the fewest predictors based on the Akaike Information Criterion (AIC) using both forward and backward predictor selection. We assessed the relative importance of each predictor with the package relaimpo (Gröemping, 2007) and calculated 95% confidence intervals using a bootstrap with 1000 replicates to test their significance. To assess for differences in the critical thermal limits of each of the three species that were collected at both elevations, we implemented a similar LMM that did not include species as a fixed factor. We assessed the significance of fixed effects using a Type II Wald χ2 test with the car package (Fox and Weisberg, 2019). When factors and factor interactions were significant, we used the lsmeans package (Lenth, 2016) to conduct multiple pairwise comparisons with Bonferroni adjustment to assess for differences among groups.

To explore the temporal variations in the temperature and humidity among ambient, control hive and experimental hives, we used the function loess in the ggplot2 package (Wickham, 2009). Temporal variations in these variables are not independent from each other, as humidity depends on temperature and conditions inside the control and experimental hives depend on ambient temperature and humidity. Thus, we used Cross-Correlation analyses (Shumway and Stoffer, 2017) using the ts and stl functions in the astsa package (Stoffer, 2014) to explore how one time series may predict or explain another. Specifically, we sought to assess how well changes in the ambient temperature and humidity are tracked by the control and experimental hives. Finally, we used a one-way ANOVA model with the lm function to assess for differences among the mean hourly values of temperature and humidity among the control hive, experimental hives and ambient conditions.

### Phylogenetic signal

To account for potential species relatedness effects on thermal tolerance, we used the time-calibrated phylogeny of stingless bees from Rasmussen and Cameron (2010) to estimate phylogenetic signal in CT_Min_ and CT_Max_. We used the function drop.tip of the ape package (Paradis *et al.*, 2004) to create a tree that only contained the species of our study or their closest relative, when they were not present. Then, we used this pruned tree to calculate the phylogenetic signal using Pagel’s λ (Pagel, 1999) with the phylosig function of phytools package (Revell, 2012). We used 10 000 simulations and a likelihood ratio test to assess for significant departure from 0 (no phylogenetic signal). Only three species occurred at both elevations and their estimates of thermal limits were similar between elevations (see results below). Thus, we used average for these species, except for the CT_Min_ of *T. angustula*, which was significantly lower at the high elevation site. To account for such a difference, we ran a test using the estimate of CT_Min_ from the high-elevation site and another one with the average CT_Min_ from both elevations.

## Results

### Ambient temperature and humidity

Temperature and relative humidity differed significantly between elevations. The mean hourly air temperature at the low-elevation (Beltrán) site was 27.7°C (± 0.08, *N* = 2046) whereas that of the high-elevation site (Tequendama) was 18.8°C (± 0.05, *N* = 2047), and such a difference was significant (Wald $\chi^{2}$ = 9049.6, *DF* = 1, *P <* 0.001). However, the magnitude of the daily variations in temperature were similar between the two elevations, ranging from 10 to 13°C of difference between the maximum and minimum values at each location (25–35°C at the low-elevation site and 15–28°C at the high-elevation site). Mean hourly air relative humidity was lower at the low-elevation site (78.1% ± 0.45, *N* = 2046) in comparison to that of the high-elevation site (93.7% ± 0.22, *N* = 2047), and that difference was also significant (Wald $\chi^{2}$ = 993.5, *DF* = 1, *P <* 0.001). However, daily changes in humidity were greater in the low-elevation site than in the high-elevation site. The difference between the maximum and minimum humidity values were always higher than 40% at the low-elevation site, reaching the lowest value at 14 h when temperature was highest. In contrast, ambient humidity was relatively constant throughout the day and night at the high-elevation site, with the difference between the maximum and minimum values always less than 20%.

### Critical thermal limits and morphological traits

**Figure 2:**
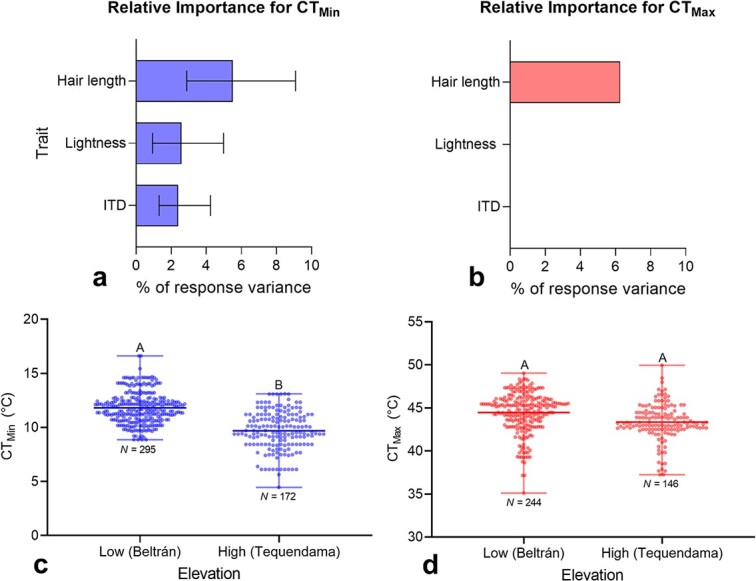
Critical thermal minima (CT_Min_) and maxima (CT_Max_) of stingless bees. (**a**, **b**) Relative importance (± 95% confidence intervals) of morphological traits for thermal limits. The best model for CT_Max_ only included hair length as a predictor. (**c**, **d**) Thermal limits and elevation. In figures (c) and (d), groups with different letters above bars are significantly different (*P* < 0.05).

ITD, hair length and lightness varied significantly among species. ITD range from 0.97 mm in *T. angustula* to 2.98 mm in *Melipona compressipes* (Fabricius), hair length from 0.04 mm in *Paratrigona eutaeniata* Camargo and Moure to 0.92 mm in *M. compressipes* and lightness from 22% in *Oxytrigona daemoniaca* Camargo to 53% in *Tetragona ziegleri* (Friese). While ITD was similar between elevations, and the interaction between species and elevation was not statistically significant, hair length and lightness differed between elevations and the interaction between species and elevation was significant (Table 1, Supplementary Table S1). On average, bees from the high-elevation site had longer hair (0.39 mm) and a darker integument (Lightness value, *L* = 27.7%) than bees from the low-elevation site (hair length = 0.31 mm; *L* = 33.2%). At each elevation, and across all species, both CT_Min_ and CT_Max_ increased significantly with increasing values of each morphological trait, except for the relationship between lightness and each thermal limit at the low-elevation site, which was not significant (Supplementary Table S2, Supplementary Fig S2). ANCOVA tests showed no significant interaction between each morphological trait and elevation, thus suggesting that the slope of regression between each thermal limit and morphological trait is similar at both elevations (Supplementary Table S3). Using Akaike’s information criterion, all three morphological traits combined resulted in the best model for CT_Min_ that explained 10.5% of its variance. The best model for CT_Max_ included only hair length and it explained 5.9% of its variance. Based on the confidence intervals, hair length and lightness are statistically more important than ITD for CT_Min_ (Fig 2a,b).

**Table 1:** Critical thermal minimum (CT_Min_) and maximum (CT_Max_), ITD and number of bee hives (N) used per species of stingless bees at two elevations in central Colombia. The mean value is followed by SE and number of individuals measured for each bioassay

Species	Low elevation (Beltrán)				High elevation (Tequendama)			
	CT_Min_, °C	CT_Max_, °C	ITD, mm	N	CT_Min_, °C	CT_Max_, °C	ITD, mm	*N*
*Cephalotrigona femorata* (Smith)	11.65 ± 0.19, *n* = 25	47.23 ± 0.16, *n* = 25	2.18 ± 0.01, *n* = 25	1	—	—	—	—
*Frieseomelitta paupera* (Provancher)	12.96 ± 0.19, *n* = 38	45.64 ± 0.23, *n* = 33	1.27 ± 0.01, *n* = 38	3	—	—	—	—
*Meliponacompressipes* (Fabricius)	—	—	—	—	11.42 ± 0.62, *n* = 10	43.95 ± 1.19, *n* = 10	2.98 ± 0.02, *n* = 10	1
*M. eburnea* Friese	—	—	—	—	10.88 ± 0.21, *n* = 30	45.30 ± 0.30, *n* = 23	2.38 ± 0.01, *n* = 30	3
*M. favosa* (Fabricius)	13.59 ± 0.37, *n* = 21	45.52 ± 0.68, *n* = 15	2.55 ± 0.02, *n* = 21	4	—	—	—	—
*Nannotrigona gaboi* Jaramillo et al.	11.62 ± 0.20, *n* = 27	43.32 ± 0.60, *n* = 21	1.34 ± 0.004, *n* = 27	2	—	—	—	—
*Oxytrigona daemoniaca* Camargo	—	—	—	—	10.68 ± 0.24, *n* = 12	43.06 ± 0.77, *n* = 11	1.36 ± 0.01, *n* = 12	1
*O. mellicolor* Packard	11.74 ± 0.23, *n* = 38	44.64 ± 0.36, *n* = 24	1.50 ± 0.01, *n* = 38	1	—	—	—	—
*Parapartamonazonata* (Smith)	—	—	—	—	7.35 ± 0.19, *n* = 38	41.82 ± 0.41, *n* = 29	1.60 ± 0.01, *n* = 38	2
*Paratrigonaeutaeniata* Camargo & Moure	—	—	—	—	10.28 ± 0.35, *n* = 9	42.97 ± 0.23, *n* = 8	1.35 ± 0.01, *n* = 9	1
*Plebeia mutisi* Engel	—	—	—	—	9.98 ± 0.19, *n* = 8	42.85 ± 0.22, *n* = 8	1.07 ± 0.01, *n* = 8	1
*Scaptotrigonamagdalenae* Engel*	10.86 ± 0.30, *n* = 24	44.03 ± 0.20, *n* = 19	1.65 ± 0.01, *n* = 24	3	10.38 ± 0.25, *n* = 16	43.14 ± 0.32, *n* = 14	1.70 ± 0.01, *n* = 16	2
*Tetragona perangulata* (Cockerell)	12.38 ± 0.31, *n* = 17	44.12 ± 0.36, *n* = 16	1.43 ± 0.02, *n* = 17	2	—	—	—	—
*T. ziegleri* (Friese)	11.48 ± 0.20, *n* = 30	44.06 ± 0.16, *n* = 28	1.26 ± 0.01, *n* = 30	3	—	—	—	—
*Tetragonisca angustula* (Latreille)*	11.34 ± 0.20, *n* = 44	43.88 ± 0.55, *n* = 31	0.96 ± 0.01, *n* = 44	4	9.78 ± 0.22, *n* = 28	43.27 ± 0.78, *n* = 24	1.00 ± 0.01, *n* = 28	3
*Trigona amalthea* (Olivier)	—	—	—	—	10.13 ± 0.50, *n* = 14	44.22 ± 0.39, *n* = 13	2.07 ± 0.01, *n* = 14	1
*T. fulviventris* Guérin-Méneville*	11.01 ± 0.16, *n* = 31	42.58 ± 0.16, *n* = 31	1.46 ± 0.01, *n* = 31	1	9.24 ± 0.27, *n* = 7	42.70 ± 0.23, *n* = 6	1.45 ± 0.01, *n* = 7	1

^*^Species found at both elevations.

### Critical thermal limits and elevation

Bees from the high-elevation site displayed a mean CT_Min_ of 9.69°C (± 0.136, *N* = 172), which is 2.2°C lower than the CT_Min_ of bees from the low-elevation site (11.81 ± 0.136, *N* = 295). CT_Min_ varied significantly across species ($\chi^{2}$ = 34.11, *DF* = 16, *P* = 0.005) and the difference in CT_Min_ between elevations was significant after accounting for body size (ANCOVA, Wald $\chi^{2}$ = 6.48, *DF* = 1, *P* = 0.011; Fig 2c). Bees from the low-elevation site displayed a mean CT_Max_ of 44.47°C (± 0.149, *N* = 146) while those from the high-elevation site showed a mean CT_Max_ of 43.34°C (± 0.175, *N* = 244). CT_Max_ also varied significantly across species ($\chi^{2}$ = 96.62, *DF* = 16, *P* < 0.001) but the difference in CT_Max_ between elevations was not significant after accounting for body size (ANCOVA, Wald $\chi^{2}$ = 1.65, *DF* = 1, *P* = 0.199; Fig 2d).

**Figure 3:**
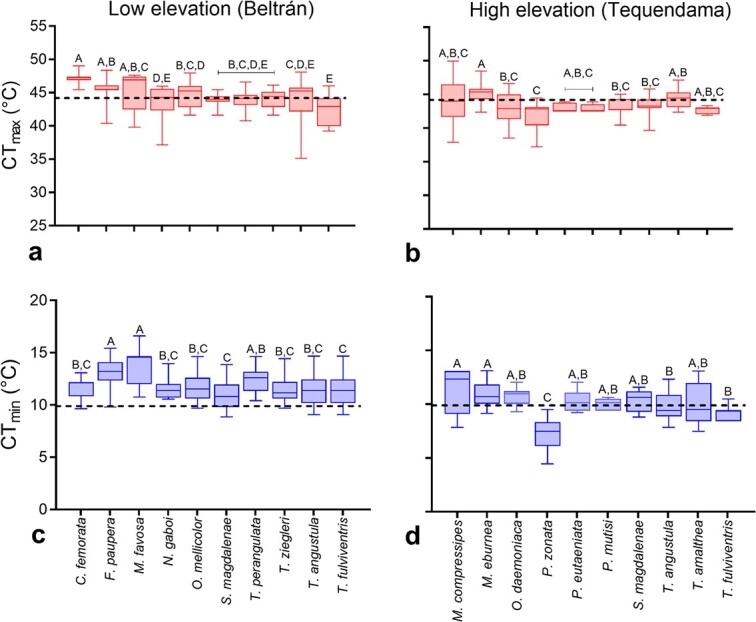
Box plots showing CT_Min_ and CT_Max_ among species of stingless bees from two elevations in central Colombia. At each elevation and for each thermal limit, groups with different letters above bars are significantly different (*P* < 0.05). To facilitate comparisons, a horizontal dashed line was placed near 10°C in plots of CT_Min_ (**c**, **d**) and near 45°C in plots of CT_Max_ (**a**, **b**).

There is a tradeoff between cold and heat tolerance among stingless bees. At each elevation, some species appeared to be more warm or cold adapted than others, as judging by their thermal limits (Fig 3, Supplementary Fig S3). For example, *F. paupera* and *M. favosa* (Fabricius) were the least cold tolerant species among the bees tested in the low-elevation site, with an average CT_Min_ of 12.96°C and 13.59°C. These two species, as well as *Cephalotrigona femorata* (Smith), displayed a CT_Max_ that was on average between 1.05°C and 2.76°C higher than the average CT_Max_ estimated for the bee community. Among the species from the high-elevation site, *Parapartamona zonata* (Smith) displayed the lowest CT_Min_ and a low CT_Max_ (Table 1, Fig. 3b,d). While an increase in CT_Min_ was not related with an increase in CT_Max_ at the low-elevation site, such a relationship was significant at the high-elevation site (Supplementary Fig S3). An ANCOVA test showed that the slope of regression between CT_Min_ and CT_Max_ is similar at both elevations (Supplementary Table S3), and a regression analysis across all species from both elevations indicated that CT_Min_ significantly increased with increasing CT_Max_ (*R^2^* = 0.24, *P <* 0.001).

Forager bees of three species, *S. magdalenae*, *T. angustula* and *T. fulviventris*, were tested at both elevations. For these species, ITD was similar between elevations for *S. magdalenae* ($\chi^{2}$ = 2.952, *P* = 0.086) and *T. fulviventris* ($\chi^{2}$ = 0.944, *P* = 0.331). However, bees of *T. angustula* from the high-elevation site were significantly larger ($\chi^{2}$ = 4.250, *P* = 0.039; *DF* = 1 in all cases, Table 1) than those from the low-elevation site. After accounting for body size, CT_Max_ was similar between elevations for each of the three species (*S. magdalenae*, $\chi^{2}$ = 0.960, *P* = 0.039; *T. angustula*, $\chi^{2}$ = 0.171, *P* = 0.680; *T. fulviventris*, $\chi^{2}$ = 0.037, *P* = 0.848; *DF* = 1 in all cases). However, after accounting for body size, CT_Min_ was similar between elevations for *S. magdalenae* ($\chi^{2}$ = 0.260, *P* = 0.610) and *T*. *fulviventris* ($\chi^{2}$ = 2.767, *P* = 0.096), but it was significantly lower for *T. angustula* at the high-elevation site than in the low-elevation site ($\chi^{2}$ = 4.742, *P* = 0.029, *DF* = 1 in all cases).

### Phylogenetic signal

Neither CT_Min_ (Pagel’s *λ* = 0.597, *P* = 1.0) nor CT_Max_ (*λ* < 0.01, *P* = 1.0) displayed significant phylogenetic signal (Supplementary Fig S4). Foragers of *T. angustula* from the high-elevation site displayed a significantly lower estimate of CT_Min_ than foragers of this species at the low-elevation site (see above). Thus, we ran another test using the estimate of CT_Min_ for foragers of *T. angustula* from the high-elevation site and found non-significant results (*λ* < 0.01, *P* = 1.0).

### Brood temperature and humidity

**Figure 4:**
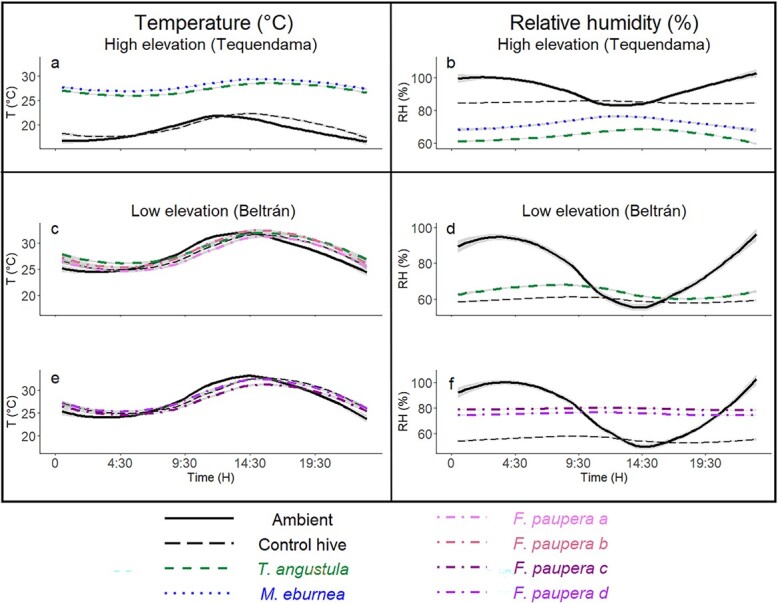
Daily changes in temperature (T, ºC) and relative humidity (RH, %) inside hives of three species of stingless bees relative to ambient conditions and unoccupied control hives at two elevations in central Colombia. (**a, b**) Temperature and humidity at the high-elevation site; (**c**–**f**) Temperature and humidity at the low-elevation site. Only hives of *Tetragonisca angustula* were available at both elevations. Due to equipment limitations, we only measured brood temperature of *Friesomelitta paupera* between February 6 and March 29 (Fig **c**), but measured both temperature and humidity between March 29 and April 20 (Figs **e** and **f**). These measurements for *F. paupera* at different dates are indicated with different colors and letters.

**Figure 5:**
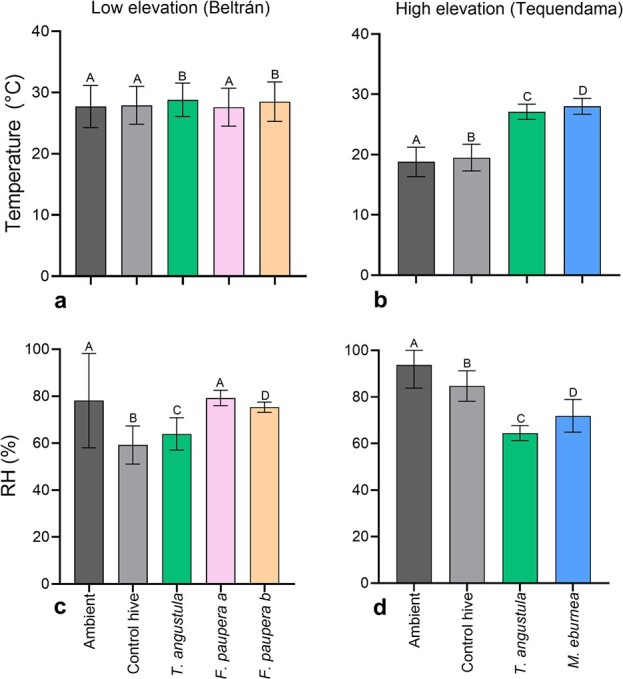
Mean hourly values (± SD) of ambient, unoccupied control hive and stingless bee nest internal temperature (°C) and relative humidity (RH, %) recorded at two elevations in central Colombia. (**a**, **b**) Temperature at the low- and high-elevation sites. (**c**, **d**) Relative humidity at the low- and high-elevation sites. At each elevation and for each variable, groups with different letters are significantly different (*P* < 0.05).

At both elevations, ambient and internal temperature of unoccupied control hives (empty wooden boxes) were either similar (low-elevation site) or significantly different but very close (high-elevation site) (Figs 4a,c,e and Fig 5a,b). However, while in the low-elevation site changes in the internal temperature of the control hive occurred within one hour after an increase or decrease of the ambient temperature (Fig 4c,e), it took about 2 h in the control hive in the high-elevation site (Fig 4a). In contrast to temperature, control hives at both elevations showed a nearly constant and lower internal humidity (59% in the low-elevation site and 85% in the high-elevation site) than ambient humidity throughout the day (Figs 4b,d,f and Fig 5c,d).

In the low-elevation site, mean hourly ambient (27.7°C ± 0.08), unoccupied control hive (27.9°C ± 0.07) and brood temperature of *T. angustula* (28.8°C ± 0.06) and *F. paupera* (27.6°C ± 0.07 and 28.5°C ± 0.07) were similar or significantly different but very close to each other (≤ 1°C higher) (Fig 5a). Brood temperature was adjusted within 1 ½ h after an increase or decrease in the ambient temperature (Fig 4c,e). In contrast, mean hourly brood temperature of *T. angustula* and *M. eburnea* in the high-elevation site was less variable throughout the day (Fig 4a) and 7.6–9.2°C higher than both ambient and control hive temperatures (Fig 5b). Mean hourly values of brood humidity were relatively constant throughout the day but varied among species and elevations with respect to ambient and control hive humidity (Figs 4b,d,f). In the high-elevation site, brood humidity of *T. angustula* and *M. eburnea* was between 20 and 30% lower than ambient and control hive humidity (Fig 5d). In the low-elevation site, while mean hourly brood humidity of *T. angustula* was 4.7% higher than the control hive and 14.2% lower than ambient humidity, that of *F. paupera* was up to 24% higher than the control hive and close to ambient humidity (Fig 5c).

## Discussion

### Critical thermal limits and elevation

Our study is the first to assess the critical thermal limits across several species and genera of stingless bees, the main group of pollinators in the tropics. We found that CT_Min_ decreased with elevation while CT_Max_ was similar between elevations. Thus, these results are partially in agreement with our expectations because CT_Max_ did not decrease with elevation. However, although unanticipated, these results are consistent with studies in other organisms (Pintanel *et al.*, 2019) including insects (Hoffmann *et al.*, 2013; Bishop *et al.*, 2017). The relatively invariant CT_Max_ is a pattern observed across a wide range of vertebrates and invertebrates, which is commonly known as Brett’s Rule or Brett’s heat-invariant hypothesis (Brett, 1956). In bees, a relatively invariant CT_Max_ has also been observed in the North American bumble bee *Bombus vosneseskii* Radoszkowski (Pimsler *et al.*, 2020), Andean bumble bees (Gonzalez *et al.*, 2022c), and honey bees (Sánchez-Echeverría *et al.*, 2019), but a decrease in CT_Max_ with increasing elevation has also been documented for other species of bumble bees (Oyen *et al.*, 2016), as well as in carpenter bees (Gonzalez *et al.*, 2020). Thus, altitudinal variations in bees’ CT_Max_ might be taxon specific.

The similar estimates of CT_Max_ between the community of bees at both elevations, which appear to be comparable to estimates of CT_Max_ of bees from higher latitudes (e.g. Hamblin *et al.*, 2017; Gonzalez *et al.*, 2020), support the idea of a conserved heat tolerance across linages (Araújo *et al.*, 2013; Sunday *et al.*, 2019). In addition to physiological constraints (Hoffmann *et al.*, 2013; Sunday *et al.*, 2019), the evolutionary history of stingless bees might also explain their conserved heat tolerance. Neotropical stingless bees evolved in the Americas about 30–40 Mya (Rasmussen and Cameron, 2010), well before the uplifting of the Colombian Andes that occurred less than 14 Mya (Gregory-Wodzicki, 2000). Thus, cooler mountain environments only recently became available to stingless bees. Our results also support the expected high vulnerability of tropical insects to global warming, particularly of those living at low elevations. While the CT_Max_ of bees from the high-elevation site is between 13°C and 17°C higher than the highest daily ambient temperature we recorded during our studies (28°C), the CT_Max_ of bees from the low-elevation site is only between 3.5°C and 8°C higher than the highest daily ambient temperature recorded (39°C). Thus, our data suggests that stingless bees from the low-elevation site are living closer to their maximum thermal limit and that mountain habitats might represent important refuge for them under global warming. Indeed, studies under climate change scenarios using niche modeling approaches predict shifts in elevation for some stingless bees to compensate for the increase in temperature (Gonzalez *et al.*, 2021). Unfortunately, the acclimation capacity of tropical insects is expected to be limited (Deutsch *et al.*, 2008; Kingsolver *et al.*, 2013), and Andean ecosystems continue to be highly threatened by deforestation, agriculture and human population growth (Armenteras *et al.*, 2011).

### Critical thermal limits and morphological traits

We found that critical thermal limits increased (high values of CT_Min_ and CT_Max_ or low cold tolerance and high heat tolerance) with increasing values of the morphological traits examined, except for the relationship between lightness and thermal limits at the low-elevation site, which was not significant (Supplementary Table S2, Supplementary Fig S2). Therefore, these results are also partially in agreement with our initial expectations because CT_Min_ did not decrease with increasing body size nor with increasing hair length, CT_Max_ did not increase with decreasing lightness (darker color), and both CT_Min_ and CT_Max_ were not related with lightness at the low-elevation site. However, although most relationships were statistically significant, these are weak and the morphological traits explained no more than 10% of the variance (Supplementary Table S2, Fig 2, Supplementary Fig. S2). Body size, hairiness and cuticular coloration are known to have a profound influence on the thermal biology of insects, including bees (e.g. Peters *et al.*, 2016; Tsai *et al.*, 2020; Buxton *et al.*, 2021). For example, studies have shown that large, light-colored bees gain and lose heat more slowly than small, dark bees. Similarly, hairy bees tend to tolerate lower temperatures than bees with short and sparse hairs (Pereboom and Biesmeijer, 2003; Peters *et al.*, 2016).

The relationships between thermal limits and body size in bees is complex, as these are not consistent among studies. While some studies indicate that both CT_Min_ and CT_Max_ increase with increasing body size in bumble bees (Oyen *et al.*, 2016), others suggest no effect of body size on heat tolerance (Hamblin *et al.*, 2017; Oyen and Dillon, 2018; Gonzalez *et al.*, 2020, 2022c). Although the increase in CT_Min_ with increasing body size and hair length was unanticipated, cold tolerance increases with decreasing body size in some species of fruit flies, at least at the population level (e.g. Poikela *et al.*, 2021). In common garden bumble bees, chill coma recovery times were longer in larger individuals, suggesting that re-establishing ion balance following cold exposure may be size-dependent (Oyen *et al.*, 2021). Thus, these results are within the range of responses documented for other insects. CT_Min_ is not measured as frequently as CT_Max_ in bee thermal studies, and the patterns documented here for stingless bees could also be displayed by other bee groups.

Body melanism is common in insects that inhabit high elevations because they are exposed to low temperatures and high ultraviolet radiation. Thus, a dark integument is hypothesized to be adaptive in these environments, as it improves passive heat gain and provides protection against solar radiation (Bishop *et al.*, 2016; De Souza *et al.*, 2020). Our results support this idea in relation to cold tolerance, as bees from only the high-elevation site with low values of lightness (darker bees) displayed low CT_Min_ (Supplementary Fig S2). The non-significant relationship between lightness and thermal limits at the low-elevation site, as well as CT_Max_ increasing with increasing lightness at the high-elevation site, suggests that body coloration might be important in other aspects and contexts of the stingless bees’ biology, such as mimicry, camouflage, foraging, resistance to pathogens and physical damage (Pereboom and Biesmeijer, 2003; Dubovskiy *et al.*, 2013) and not necessarily related with their thermal limits. In addition, the negative effects of temperature extremes might occur before the thermal limits are reached. Thus, future studies should assess the influence of body coloration using other metrics of thermal tolerance.

Our study indicates that ITD and lightness are predictors that are not as important as hair length (Fig 2a,b). This suggests that other factors, including other morphological or functional traits or even other aspects of the assessed morphological traits might be more relevant to stingless bees’ thermal limits. For example, a study in North America (Hamblin *et al.*, 2017) suggests that estimates of bees’ thermal limits might be influenced by life history traits. Based on that study, eusocial bees display a higher CT_Max_ than solitary species, while cavity-nesting bees display a lower CT_Max_ than stem or ground-nesting species. Our estimates of stingless bees’ CT_Max_ are equally high to that of other eusocial bees, such as bumble bees and honey bees (Oyen *et al.*, 2016; Hamblin *et al.*, 2017; Sánchez-Echeverría *et al.*, 2019 ; Gonzalez *et al.*, 2022a,c). These estimates are also higher than most solitary bees we tested during our field work, except for carpenter bees that displayed a higher CT_Max_ (Gonzalez *et al.*, unpublished data). To date, CT_Min_ has only been assessed in bumble bees (Oyen *et al.*, 2016; Oyen and Dillon, 2018; Pimsler *et al.*, 2020; Maebe *et al.*, 2021; Gonzalez *et al.*, 2022c) and honey bees (Sánchez-Echeverría *et al.*, 2019; Gonzalez *et al.*, 2022a). While estimates of CT_Min_ for honey bees are within the range of those we estimate for stingless bees, those of bumble bees tend to be significantly lower (< 6°C) than estimates for either honey or stingless bees. This is not surprising given that bumble bees are known for being largely cold-adapted species. In addition, information on bumble bees’ thermal limits is from species occurring in temperate areas where they experience much lower temperatures than in the tropics. Furthermore, other morphological traits might be more informative for stingless bees’ thermal limits. For example, water content, cuticle thickness and sculpturing, hair density and color, type of hair (simple and erect vs. branched and decumbent) and color of metasoma. Further studies should explore them, as some of these traits influence ants’ thermal limits (Buxton *et al.*, 2021).

In this study, we were able to recognize some species that are warm (*C. femorata*, *F. paupera*, *M. favosa*) or cold (*P. zonata*) adapted based on their high CT_Max_ or low CT_Min_. Our analyses indicated that performance at low and high temperatures might be inversely correlated, as bees tended to be either cold or heat tolerant but not both (Supplementary Fig S3). This suggests that adaptations to favor either cold or heat tolerance in stingless bees are costly, which might limit their potential for adaptation to changes in temperature (e.g. Schou *et al.*, 2022). The two warm-adapted species are common in tropical dry forests while the single cold adapted species in our study is among the few stingless bees that are restricted to montane environments in South America. There is nothing outstanding about the nesting biology or morphology of these species that make them prone to experience a different thermal environment than that of other sympatric species. The warm-adapted species vary from small (*F. paupera*) to large (*M. favosa*) in body size, they all nest inside empty cavities, and they build an involucrum surrounding the brood area, except for *F. paupera*. The cold-adapted species, *P. zonata*, is relatively small, nests in the ground and builds an involucrum. Thus, the high heat or cold tolerance displayed by these bees might be driven by genetic mechanisms and tied to aspects of local climate, as documented for bumble bees (Pimsler *et al.*, 2020), rather than differences in their life history traits. The high heat or cold tolerance might also be constrained across the phylogeny, a pattern that has been described in fruit flies (Kellermann *et al.*, 2012). However, our analysis suggests that there is no phylogenetic signal in our estimates of CT_Min_ and CT_Max_. Doubtless, the lack of any statistically significant signal in our study is probably due to the small number of taxa we used, as well as the small geographical scale of our study, as reported in some studies with ants (Nascimento *et al.*, 2022). Thus, future work should address this by including more species and representatives from other linages.

It is important to note that the critical thermal limit estimates reported in this study are likely the result of both genetic and environmental effects on the phenotype (Angilletta, 2009). Thus, we do not know if the apparent differences between elevations and among species are due to plastic responses (acclimation) or genetic differences (local adaptation). Future studies rearing bees under a common garden design (common laboratory conditions) or transplant experiments between elevations are necessary to control for plastic effects that may confound species comparisons (Kellermann *et al.*, 2012; Kellermann and van Heerwaarden, 2019). In addition, we conducted our study in a narrow temporal window (dry season) and seasonal variations in temperature are known to influence ants’ thermal limit estimates (Bujan *et al.*, 2020). We also used bees from a small number of populations and, in some cases, from a reduced number of individuals taken from a single nest (Table 1). Thus, future studies should focus on assessing variations in stingless bees’ thermal limits at different periods of the year and from different populations and nests. Despite these limitations, the results of our experiments suggest that thermal tolerance traits are likely a good metric for determining the vulnerability of stingless bees to climate change as they are influenced by both physiological and morphological traits and vary across environmental gradients.

### Brood temperature and humidity

Brood temperature and humidity has been assessed in a few stingless bee species. Average brood temperature ranges from 25°C to 35°C (Roubik and Aquilera, 1983; Solarte *et al.*, 2015), with some species experiencing the highest mortality at ≤ 22°C and ≥ 38°C (Vollet-Neto *et al.*, 2015; Araújo *et al.*, 2017). The average brood temperatures recorded in our work (27–29°C) are within the range of brood temperatures documented for other species, and, together with published records, suggest significant variations in the thermal environment in which immature stingless bees develop. If the thermal environment during immature stages influences the adult phenotype (Kellermann and van Heerwaarden, 2019), it is possible to expect differential plastic responses in thermal tolerance among species of stingless bees. Indeed, honey bees reared at 20°C improved their cold tolerance in comparison to bees that were reared at 24°C or 34°C (Sánchez-Echeverría *et al.*, 2019). Based on the nest architecture alone, stingless bees that nest in empty cavities seem to have similar abilities to regulate brood temperature and humidity regardless of the presence of an involucrum and cell arrangement, although we only tracked one or two nests of each species. Future studies should assess internal nest conditions of stingless bees with other nesting biology, such as species building aerial nests or nests in the ground.

Brood temperature from hives at the low-elevation site tracked ambient temperature closely (Figs 4c,e), while hives from the high-elevation site maintained a more stable and higher temperature (Fig 4a). In contrast to temperature, brood humidity was more uniform throughout the day, regardless of the elevation (Figs 4b,d,f). Thus, these observations suggest that while bees regulated brood humidity, they either thermoconformed at low elevation (Figs 4c,e) or thermoregulated at high elevation (Fig 4a). It has long been known that stingless bees are generally poor thermoregulators in comparison to honey bees and, that for some species, the regulation of humidity is sometimes more important than temperature (Torres *et al.*, 2007, 2009; Halcroft *et al.*, 2013). However, our exploratory study is the first in shedding light on how malleable these behaviors can be in relation to changes in temperature across elevation. Bees thermoconformed where fluctuations in ambient temperature are optimal or within the range of tolerable temperatures for colony development, whereas they thermoregulated where conditions are outside of optimal range. This is clear in *T. angustula*, the single species in our study with available hives at both elevations. However, *F. paupera*, the other species at our low-elevation site, might also have the same response at higher elevations. Torres *et al.* (2009) documented nest thermoregulation by this species at 1200 m in northern Colombia, which agrees with our observations on *T. angustula*. Social behavior is expected to provide insects with a greater behavioral plasticity to tolerate or to adapt to changes in climate (Parr and Bishop, 2022), and our observations illustrate these potential social responses to climatic variability across the elevation gradient.

The regulation of humidity inside the hive, regardless of the elevation, is another significant result in our study. Although immature bee stages have a higher desiccation risk than adults, regulation of humidity inside social nests has not yet received the same attention as that of temperature (Ellis *et al.*, 2008). Most studies do not assess hive humidity and the few studies indicate that hygroregulation, at least for some species, is even more important than thermoregulation for colony health (Solarte *et al.*, 2015; Ayton *et al.*, 2016). Understanding stingless bees’ responses to changes in humidity at the colony level should also be included in future thermal studies considering that changes in rainfall patterns are predicted to be more drastic near the equator than at other latitudes (IPCC, 2013).

### Implications for conservation

Our results have significant implications for the conservation and sustainable use of stingless bees. We showed that bees have differential thermal sensitivities, as judging by their critical thermal limits, and thus not all species are going to be affected the same way to heat or cold stress. We showed that species or populations might be climatically adapted, so that bees from high elevations can handle lower temperatures than those from low elevations. In addition, our work suggests that bees from low elevations are living closer to their maximum thermal limit than those from high elevations. This means that some species might be more vulnerable to global warming than others, which matches the differential responses predicted under climate change scenarios for stingless bees (Gonzalez *et al.*, 2021). Although more studies are necessary to determine if differences in stingless bees’ thermal limits are due to plastic responses or genetic differences, at least one study suggests that some tropical insects display very limited phenotypic plasticity (García-Robledo and Baer, 2021). From a practical standpoint, these results provide additional support to current concerns related to the long-distance relocation of wild nests, especially across the altitudinal gradient. For example, based on the elevational difference in CT_Min_ displayed by *T. angustula* in our study, one might predict negative effects if nests are relocated from low to high elevations, even within a 1.5 km change in altitude. Indeed, documented cases of long-distance relocations of nests of this species in Colombia, both across elevations and ecosystems (e.g. dry vs. humid forests), have already resulted in total colony loss or low establishment of nests. *Tetragonisca angustula* is the most common species of stingless bees used in meliponiculture in Colombia and an informal market of hives from low elevation areas, where the species is abundant, to mid elevation areas where agricultural production is high, is increasingly common. Local adaptations to other environmental conditions besides temperature, such as humidity, might be also important for stingless bees. Unfortunately, the desiccation tolerance of stingless bees, or bees in general, remains to be explored.

## Funding

This work was supported by a Fulbright Colombia-National University of Colombia Distinguished Chair in Biodiversity and Sustainable Development award, Fulbright U.S. Scholar Program, National University of Colombia’s Office of Research (Hermes #51171), as well as the University of Kansas’ Center of Latin American and the Caribbean Studies, and National Science Foundation (DBI 1560389, DBI 2101851).

## Data availability

The data underlying this article are available in the article and in its online supplementary material.

## Supplementary Material

Web_Material_coac073
