# Supplementary material for: Neotropical stingless bees display a strong response in cold tolerance with changes in elevation
Source: Conserv Physiol. 2022 Dec 21;10(1):coac073. doi: 10.1093/conphys/coac073 (PMC9773376; doi:10.1093/conphys/coac073)

Supplemental Materials

**Neotropical stingless bees display a strong response in cold tolerance with changes in elevation**

Victor H. Gonzalez^1*^, Kennan Oyen^2^, Nidya Vitale^3^, and Rodulfo Ospina^4^

^1^Undergraduate Biology Program and Department of Ecology and Evolutionary Biology, University of Kansas, Lawrence, Kansas, 66045, U.S.A.

^2^Department of Biological Sciences, McMicken College of Arts and Sciences, University of Cincinnati, 318 College Drive, Cincinnati, OH 45221

^3^Instituto Argentino de Investigaciones de las Zonas Áridas, CONICET, Mendoza, Argentina.

^3^Laboratorio de Investigaciones en Abejas, Universidad Nacional de Colombia, Santa Fé de Bogotá, Colombia

*Corresponding author: Undergraduate Biology Program and Department of Ecology and Evolutionary Biology, University of Kansas, Lawrence, Kansas, 66045, U.S.A.

Email: vhgonza@ku.edu

**Table 1S.** Summary of results from linear mixed-effect models to examine differences in intertegular distance, hair length and Hue among species and between elevations. Wald *X^2^* value is followed by *P*-value (significant values in boldface).

| **Source** | **DF** | **ITD (mm)** | **Hair length (mm)** | **Lightness** |
| --- | --- | --- | --- | --- |
| Species | 16 | 10592.89, *P <***0.001** | 5.22, *P <***0.001** | 9.79, *P <***0.001** |
| Elevations | 1 | 2.81, *P* = 0.09 | 1.39, *P <***0.001** | 7.91, *P <***0.001** |
| Species × Elevations | 2 | 2.13, *P* = 0.34 | 2.08, *P <***0.001** | 1.42, *P <***0.001** |

**Table 2S.** Summary of results from linear regression analyses to explore the relationship between each critical thermal limit (CT_Min_ and CT_Max_) and each morphological trait per elevation. Adjusted R-squared value followed by P-value (significant values in boldface).

| **Thermal limit/Elevation** | **ITD (mm)** | **Hair length (mm)** | **Lightness** |
| --- | --- | --- | --- |
| **CT_Min_** |  |  |  |
| Low (Beltran) | 0.04, *P <***0.001** | 0.12, *P <***0.001** | -0.003, *P =* 0.94 |
| High (Tequendama) | 0.11, *P <***0.001** | 0.19, *P <***0.001** | 0.07, *P <***0.001** |
| **CT_Max_** |  |  |  |
| Low (Beltran) | 0.09, *P <***0.001** | 0.061, *P <***0.001** | -0.003, *P =* 0.66 |
| High (Tequendama) | 0.13, *P <***0.001** | 0.18, *P <***0.001** | 0.08, *P <***0.001** |

**Table 3S.** Summary of results from mixed-model ANCOVA that compare the slope of regression between elevations for each morphological trait and critical thermal limit (CT_Min_ and CT_Max_), and between CT_Min_ and CT_Max_. Wald *X^2^* value is followed by *P*-value (significant values in boldface).

| **Source** | **DF** | **ITD (mm)** | **Hair length (mm)** | **Lightness** | **CT_Max_ (ºC)** |
| --- | --- | --- | --- | --- | --- |
| **CT_Min_** |  |  |  |  |  |
| Species | 16 | 37.06, *P*=**0.02** | 0.21, *P =*0.07 | 43.63, *P <***0.001** | 50.389, *P <***0.001** |
| Elevation | 1 | 6.38, *P* = **0.01** | 25.12, *P*=0.19 | 5.72, *P =* **0.02** | 7.20, *P <***0.001** |
| Trait × Elevation | 1 | 3.49, *P* = 0.06 | 0.96, *P=*0.33 | 0.09, *P=* 0.77 | 1.98, *P =* 0.16 |
| **CT_Max_** |  |  |  |  |  |
| Species | 16 | 72.93, *P <***0.001** | 56.82, *P <***0.001** | 79.23, *P <***0.001** | — |
| Elevations | 1 | 1.64, *P* = 0.20 | 1.11, *P* = 0.29 | 2.06, *P* = 0.15 | — |
| Species × Elevations | 1 | 0.02, *P* = 0.88 | 0.35, *P*= 0.56 | 0.69, *P* = 0.41 | — |

**Figure 1S**. Study areas in Colombia. Google Earth, earth.google.com/web/.


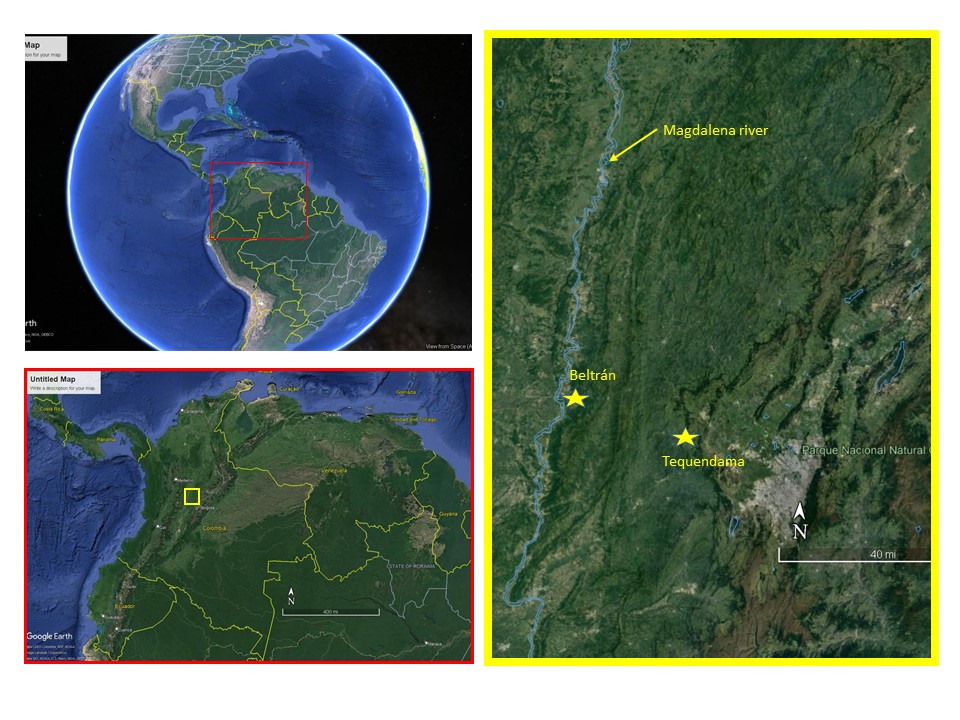


**Figure 2S.** Critical thermal minima (CT_Min_) and maxima (CT_Max_) of stingless bees and their relationship with the examined morphological traits at each elevation. a, b, intertegular distance (ITD); c,d, hair length; e,f, lightness value (0 = black, 100 = white). See Tables 2S and 3S for adjusted R-squared values for each regression and results from tests of homogeneity of regression slopes between elevations.


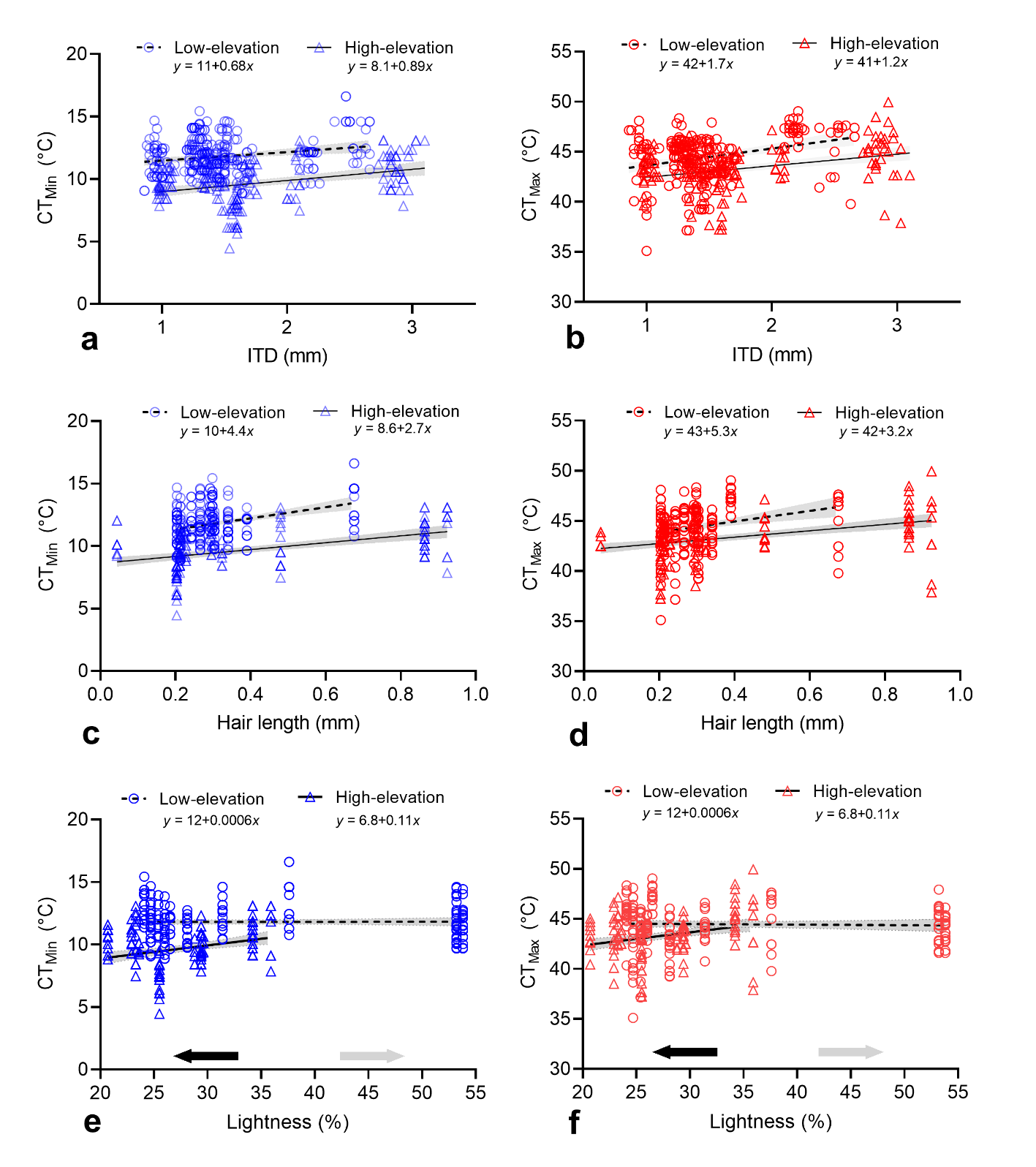


**Figure 3S.** Relationship between critical thermal minima (CT_Min_) and maxima (CT_Max_) of stingless bees at each elevation. See Table 3S for results from test of homogeneity of regression slopes between elevations.


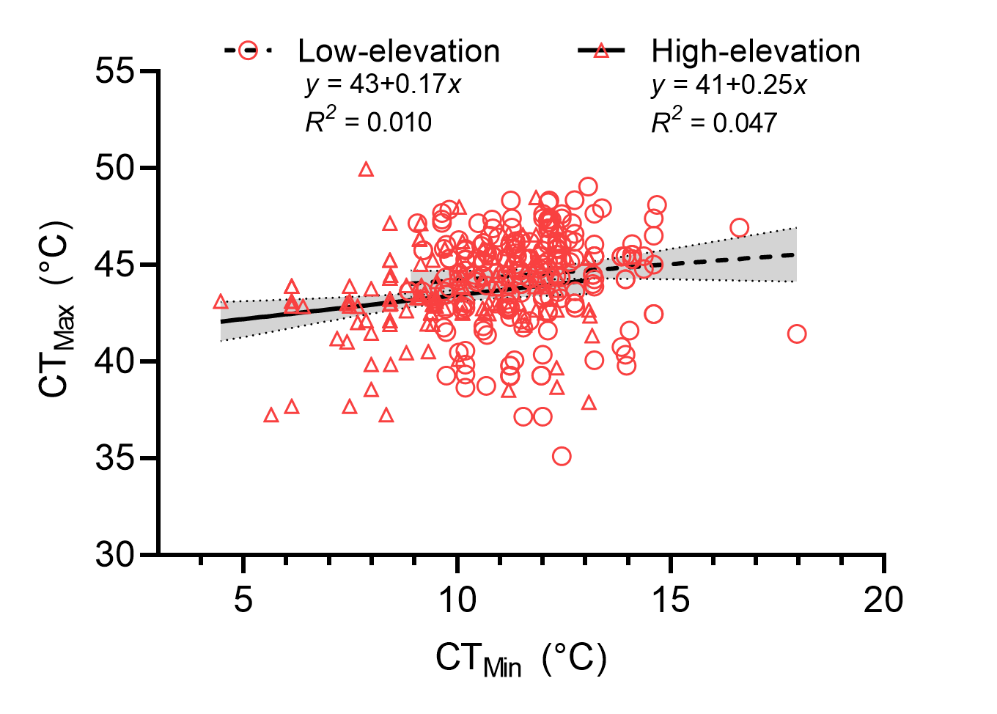


**Figure 4S.** Phylogenetic reconstruction of the focal stingless bee species used in this study and their critical thermal minima (CT_Min_) and maxima (CT_Max_). Phylogeny modified from Rasmussen and Cameron (2010).


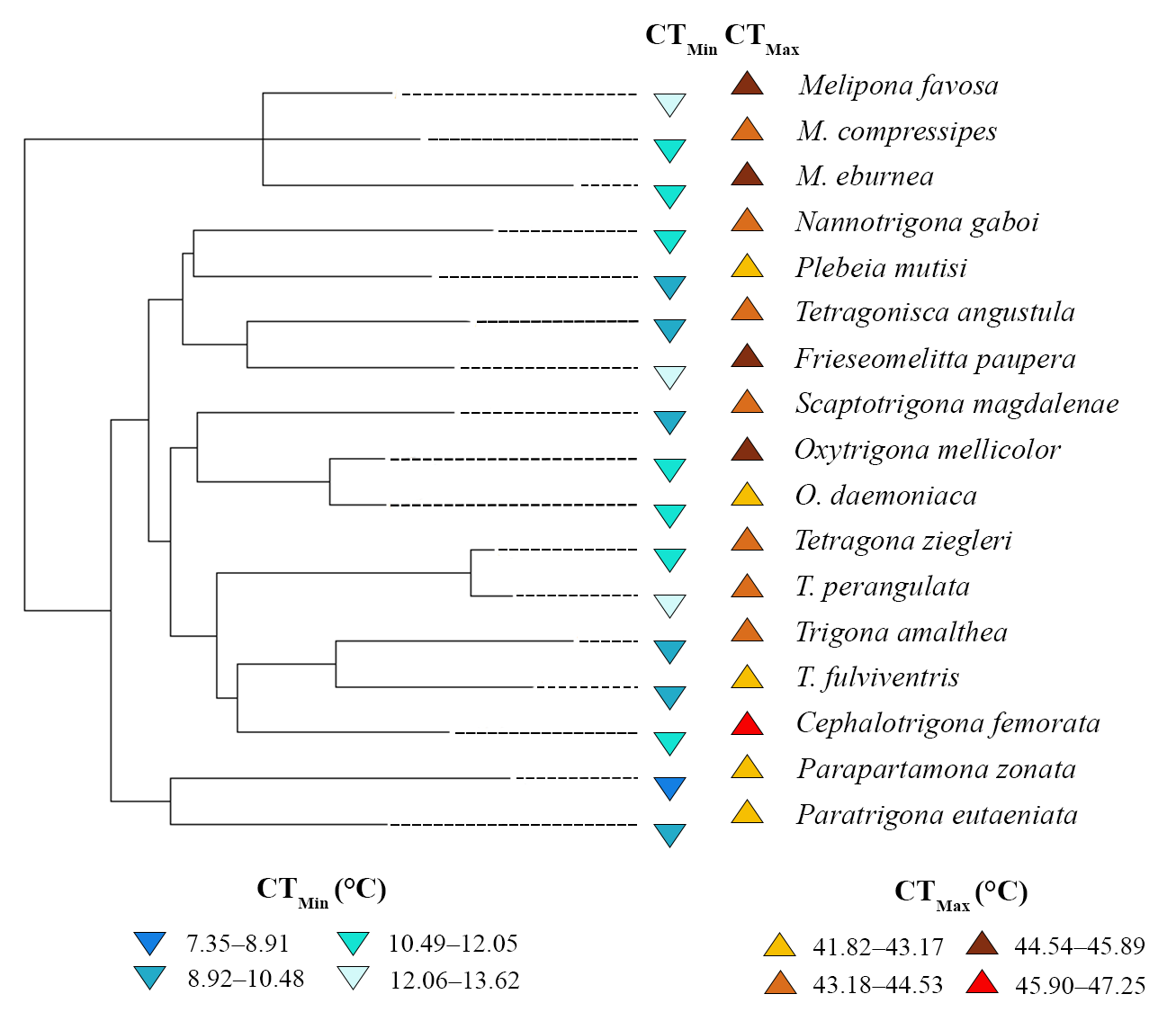

Supplement: Web_Material_coac073 [file web_material_coac073.zip › Meliponini Supp Materials_Revised_10_24_2022_CLEAN.docx]
